# Genetics of colorectal cancer

**Published:** 2014

**Authors:** I Munteanu, B Mastalier

**Affiliations:** * Department of General Surgery, CF-2 Hospital, Bucharest, Romania; ** ”Carol Davila” University of Medicine and Pharmacy, Surgery Clinic No. 2, Colentina Clinical Hospital, Bucharest, Romania

**Keywords:** colorectal cancer, oncogenes, k-ras mutations

## Abstract

The occurrence of colorectal cancer is related to the interaction that takes place at several levels between hereditary factors, environmental and individual ones. Understanding the molecular basis is important because it can identify factors that contribute to the initiation of development, maintenance of progression but also determine the response or resistance to antitumor agents. Understanding colorectal cancer at the molecular level has provided data used for genetic tests of family forms, it defined predictive markers used to select patients susceptible to certain forms of therapy and also for the development of molecular diagnostic tests to detect early non-invasive cancers.

**Abbreviations:** CIN = chromosomal instability; MMR = mismatch repair genes; MSI = Microsatellite instability, HNPCC = hereditary nonpolyposis colorectal cancer, NSAID’s = nonsteroid anti-inflammatory drugs

The elucidation of the human genome sequencing has made it possible to identify genetic alterations in cancer in an unprecedented detail. For a systematic analysis of such alterations, the sequence of human protein coding genes with a well-defined role was decisive. Colorectal cancer has a high genetic heterogeneity, which makes it difficult to determine the clinical consequences of individual mutations. It has been shown that some considered that rare mutations in colorectal cancer are actually quite common and may be involved in the occurrence of other types of cancer [**1**,**2**]. This data set new targets for the diagnosis and therapeutic interventions and opened new pathways in tumor biology research [**3**]. The genome stability is essential in maintaining cellular integrity. The loss of genomic stability leads to colorectal cancer progression through the acquisition of new mutations associated with tumor phenotype. During the past 15 years multiple genetic alterations affecting genes that control cell maturation and growth have been identified, confirming the genetic role in the occurrence of cancer [**26**,**27**].

The following types of genomic instability are described:

-Chromosomal instability 

- Microsatellite instability

-Aberrant DNA-methylation

**Chromosomal instability**

It is the most common form of genomic instability and leads to many changes in the number and chromosome structure. Chromosomal instability leads to loss of the wild allele of suppressor genes such as APC, P 53, SMAD 4, that normally prevent the occurrence of the malignant phenotype [**4**,**5**]. Although most colorectal cancers show chromosomal instability (CIN), only a few genes that cause this phenotype have been identified and did not result in any general mechanism to underpin their operation. The sequence of 102 human homologues from 96 known genes was decisive in the process of systematically identifying somatic mutations in genes with CIN potential in colorectal cancers. 11 somatic mutations have been identified over the five genes in a batch that included 132 cases of colorectal cancer. Afterwards, it was demonstrated that these mutations result in chromosomal instability and defects of chromatin cohesion in human cells [**6**]. Molecular events resulting from chromosomal instability underlie the initiation, promotion and tumor progression. This process involves environmental factors, hereditary and acquired somatic mutations in colorectal epithelium.

**Microsatellite instability**

Studying mating errors in DNA bases in patients with colorectal cancer has observed that genes responsible for repairing were inactive. Those genes were called DNA mismatch repair genes - MMR. The inactivation can be inherited (hereditary non-polyposis cancer), or acquired. Loss of DNA mismatch repair function is associated with the so-called microsatellite instability phenomenon. Microsatellite instability – MSI, refers to the change in the number of mono, bi -, tri-and tetraploid nucleotide which normally repeats in genomic DNA (microsatellites) or in the transcription of proteins [**7**]. Mutations of MLH 1, MSH 2 MSH 6 and PMS 2 genes lead to the development of Lynch syndrome, increasing cancer susceptibility [**8**]. Most of these cancers occur in the proximal colon, in elders and are often associated with women. A frequently concomitant inactivation of tumor suppressor genes is observed in these patients [**9**]. Every year, more than one million patients will develop colorectal cancer and 3% of them will have Lynch syndrome, predisposing these patients to develop HNPCC. Genetic instability dramatically emphasizes the rhythm of cancer development in these patients, reporting cases of colorectal cancer that was developed in 36 months after a negative colonoscopy [**10**]. In 70-80%, the location is proximal to the splenic flexure and the average age at which cancer develops is 45. Thus, colonoscopy in these patients is annually indicated or at every 2 years starting from the age of 25 until 40 and annually over the age of 40. Given the high risk of synchronous injuries and / or metachronous RCC in these patients, a subtotal colectomy may be required. Also, because 40-60% of female patients are at risk of developing endometrial cancer a prophylactic hysterectomy is recommended [**7**,**8**,**10**].

**Aberrant DNA methylation**

Methylation of the cytosine in the fifth position of the pyrimidine ring is a common alteration in mammals at CpG sequences. In normal cells, CpG islands are unlimited, while sporadic CpG dinucleotides are methylated in the rest of the genome. There is a gradual reversal of the profile of methylation that leads to CpG islands methylation and the loss of global methylation level during aging; this change is also very pronounced, during the process of carcinogenesis. A decrease in cytosine methylation and an aberrant considerable methylation of CpG islands associated with some promoters is found in colorectal cancer. The somatic epigenetic inactivation blocks the expression of MLH 1, in sporadic colorectal cancer with satellites instability. The molecular mechanism remains unknown but the phenomenon has been repeatedly observed in about 15% of patients with colorectal cancer and it is present in almost all tumors with aberrant methylation of MLH 1 [**11**].

**Tumor progression**

The occurrence and development of colorectal cancer remains among the most eloquent evidence of cancer in stages. The sequence of adenoma to carcinoma transformation is based on the acquisition of mutations that lead to the promotion of tumor phenotype by the selection of variants with best survival, growth and invasion of the colonies of cancer cells [**5**].

**Tumor suppressor genes and oncogenes associated with colorectal cancer**

Oncogenes are genes whose expression is intimately associated with cell normal cells transformation to cancer cells. Tumor suppressor genes: are genes that code the synthesis of proteins with a role to maintain the function of a normal cell. Oncogenes with a proven role in colorectal cancer are Ras, EGFR (Erb-B1), Erb - B2, TGFalfa, TGF-beta 1. Suppressor genes are APC, p 53, p27, MSI, LOH 18q, deletion 5 q allele, DNA Hyper methylation.

**Ras**

Ras gene mutations have been reported in 40-50% of all colorectal cancers [**12**,**13**]. Ras family of oncogenes encodes the proteins of the plasmatic membrane at its inner surface that bind guanine nucleotides and has GTP–azis activity. Ras oncogenes produce trigger signals for cell proliferation, being intimately involved in the cell cycle, which is believed to be an early event in colorectal tumor genesis [**14**]. K-ras mutations were studied to determine their role in the predictability of response to chemotherapy treatment; Thus, in patients with colorectal tumors and K-ras mutations a worse response to adjuvant treatment with 5 - FU was observed compared with groups of patients who did not have this mutation [**15**,**16**].

**APC gene** signaling is inappropriately activated; APC acts as a brake for beta-catenin [**17**]. The loss of function of the APC gene mutation is the most common mutation in colorectal cancer. In the absence of APC, Wnt gene is illustrated by the autosomal dominant condition, familial adenomatous polyposis (FAP), in which hundreds to thousands of adenomatous colonic polyps develop, leading to almost 100% lifetime risk of developing CRC in the absence of pre-emptive colectomy. 

**TP 53 Gene**

It is a tumor suppressor gene known as the “guardian of the genome” knowing its frequent damage in solid cancers. Located on chromosome 17 and present in 50% of the sporadic colorectal cancers, it facilitates carcinogenesis [**15**]. Regarding the role of p53 status in response to therapy, the study of homozygous cell lines for p 53 mutation showed a high degree of resistance to radiotherapy and some chemotherapies including 5 - FU [**18**].

**Table 1:** Tumor-Suppressor Genes and Oncogenes Commonly Associated with Colorectal Cancer (Molecular Basis of Colorectal Cancer: Sanford D. Markowitz, M.D., Ph.D., and Monica M. Bertagnolli, M.D.)

Affected Gene	Frequency %	Nature of Defect	Comments
APC	85	Activation of Wnt signaling due to the inability to degrade the β-catenin oncoprotein. Inactivating mutation causes loss of regulation of spindle microtubules during mitosis .	Germ-line mutation in familial adenomatous polyposis; somatic inactivation found in 85% of sporadic colorectal cancers. APC mutations cause chromosomal instability.
MLH1, MSH2, MSH6, PMS2	15–25	DNA single-nucleotide mismatch-repair defect permitting the accumulation of oncogenic mutations and tumor-suppressor loss. Inactivating mutation impairs ability to repair strand slippage within nucleotide repeats.	Germ-line mutation in hereditary nonpolyposis colorectal cancer; epigenetic silencing causes loss of tumor MLH1 protein expression. MMR gene mutations cause microsatellite instability.
TP53	35–55	5 Encoding a protein responsible for cell-cycle regulation inactivating missense mutations paired with loss of heterozygosity at 17p. Inactivating mutation causes loss of regulation of cell-cycle arrest and cell death.	Germ-line mutation in Li–Fraumeni syndrome. Inactivation may coincide with malignant transformation of adenomas.
TGFBR2	25–30	Receptor responsible for signaling pathways mediating growth arrest and apoptosis; inactivated by frame shift mutation in polyA repeat within TGFBR2 coding sequence in patients with mismatch-repair defects or by inactivating mutation of kinase domain.	Mutation present in >90% of tumors with microsatellite instability and 15% of microsatellite stable colon cancers
KRAS	35–45	Encoding the KRAS G-protein, with constitutive activation resulting in the activation of both the PI3K–PDK1–PKB and RAF–MEK–ERK1/2 signaling pathways, thereby promoting cell survival and apoptosis suppression.	Germ-line mutation in the cardiofaciocutaneous Syndrome. KRAS mutation occurs as early event in adenoma-carcinoma sequence: concordance of primary tumor and metastases.
SMAD4	10–35	Critical components of transforming growth factor β pathway signaling, along with related proteins SMAD2 and SMAD3; SMAD4 and SMAD2 are located on chromosome 18q, a frequent site of loss of heterozygosity in colorectal cancers; inactivated by homozygous deletion or mutation.	Germ-line mutations in familial juvenile polyposis, with a risk of colorectal cancer as high as 60% over three to four decades.
PTEN	10–15	Promotion of the activation of PI3K pathway signaling through the loss of function by inactivating mutation, resulting in cell-survival signaling and apoptosis suppression.	Germ-line mutation in Cowden’s syndrome, which carries a high risk of breast cancer, with 10% increased risk of colorectal cancer; possible role in maintenance of chromosomal stability
BRAF	8–12	Activating mutation in the BRAF serine–threonine kinase, a downstream mediator of signaling through the RAF–MEK–ERK1/2 pathway, which mimics the biologic consequences of KRAS mutation	Associated with hyperplasic polyposis, with increased incidence in serrated adenomas, like KRAS, germ-line mutation in the cardiofaciocutaneous syndrome

**Other changes in tumor cell biology**

**Aberrant regulation of signaling with prostaglandins**

The activation of growth factors is common in colorectal cancer. An essential step in the development of adenomas is prostaglandin signaling. COX-2 is an enzyme that mediates prostaglandin E2 synthesis associated with colorectal cancer. In about two thirds of colorectal cancer, COX level was increased [**19**]. Clinical trials have shown that COX - 2 inhibitions by NSAID’s - nonsteroid anti-inflammatory drugs prevent the development of new adenomas [**20**-**22**].

**Epidermal growth factor receptor**

Epidermal growth factor receptor, also known as EGFR, ErbB-1, HER 1 is a soluble protein, a tyrosine kinase with effects on the intestinal cell torpidity. EGFR exists on the cell surface and is activated by ligation with various ligands, including epidermal growth factor. Genetic mutations that lead to EGFR overexpression were associated with cancer, mainly lung and colorectal cancer. Clinical data have shown that colorectal cancers with this mutation do not respond to anti-EGFR therapy [**23**,**24**].

**Vascular growth factor**

Vascular growth factor - VEGF is responsible for the appearance new formation vessels, angiogenesis. The fatal evolution of colorectal cancers is closely related to this factor. The treatment with VEGF antibody (bevacizumab) increased the survival of patients compared to patients treated with standard therapy [**25**].

An important transposition into medical practice of colorectal cancer genetics data is the developing of molecular diagnosis in order to detect cancer in early stages. Techniques have been developed to detect specific mutations of colorectal cancer and aberrant DNA methylation in DNA extracted from feces of patients with colorectal cancer or advanced adenomas, which have a sensitivity detection of cancer in its early stages by 46-77% (72% in stage I / II, 43.7% in stage III / IV). Usually, multitarget panels that detect mutations in the APC gene, p53, K-ras, BAT-26 (a marker for microsatellite instability) are used and a marker of abnormal apoptosis [**26**,**27**]. Genetic epidemiology studies and those on monozygotic twins have shown that 35-100% of adenomas and colorectal cancers develop in individuals with inherited susceptibility. In addition, there are some families with a syndrome similar to HNPCC without evidence of repair gene mutation of DNA bases mismatch [**28**-**30**].

## Conclusions

The studies, which contribute to the understanding of colorectal cancer at the molecular level, have provided data used for genetic tests of family forms, defining predictive markers for the selection of patients susceptible of certain forms of therapy and the development of molecular diagnostic tests for the detection of early non-invasive cancers.

New biological pathways have been identified that may lead to the discovery of new therapeutic agents. Although some high-frequency mutations are attractive targets for the development of new drugs, they could cover targets located downstream on common signaling pathways.

Understanding the signals that dictate the metastatic phenotype will provide necessary information to develop new drugs to prevent and control disease progression/advanced disease.
